# Torticollis as a sign of spinal tuberculosis

**DOI:** 10.11604/pamj.2020.36.277.22977

**Published:** 2020-08-14

**Authors:** Rim Boussetta, Mohamed Zairi, Sami Bouchoucha Sami, Rafik Lafrem, Ahmed Msakeni, Walid Saied, Nebil Nessib

**Affiliations:** 1 Department of Orthopaedic Surgery, Université El Manar, Faculty of Medicine of Tunis, Tunis, Tunisia,; 2 Children's Hospital, Pediatric Orthopedic Surgery Department, Béchir Hamza, Tunis, Tunisia

**Keywords:** Tuberculosis, cervical, spine, apophysis

## Abstract

Bone localization of tuberculosis mainly affects the thoracolumbar spine. The cervical spine is rare. Its diagnosis is often late which exposes to great instability and potentially serious complications. We report the case of a 12-year old girl with no medical history, showing torticollis and high temperature without neurological complication. In the physical examination, he had torticollis and pain in the third, fourth and fifth cervical vertebra. When the biopsy was performed, we find an inter apophysis (between C7 and D1) collection. The histological examination confirmed the diagnosis of apophysis tuberculosis. The management based on tuberculosis chemotherapy and immobilization started as soon as possible.

## Introduction

Tuberculosis spine involvement is the most common site after pulmonary localization. Neurological complications and orthopaedics deformation represent the severity of this affection. Bone localization of tuberculosis mainly affects the thoracolumbar spine and usually interests the vertebral body. Isolated posterior arch involvement is exceptional.

## Patient and observation

**Figure 1:**
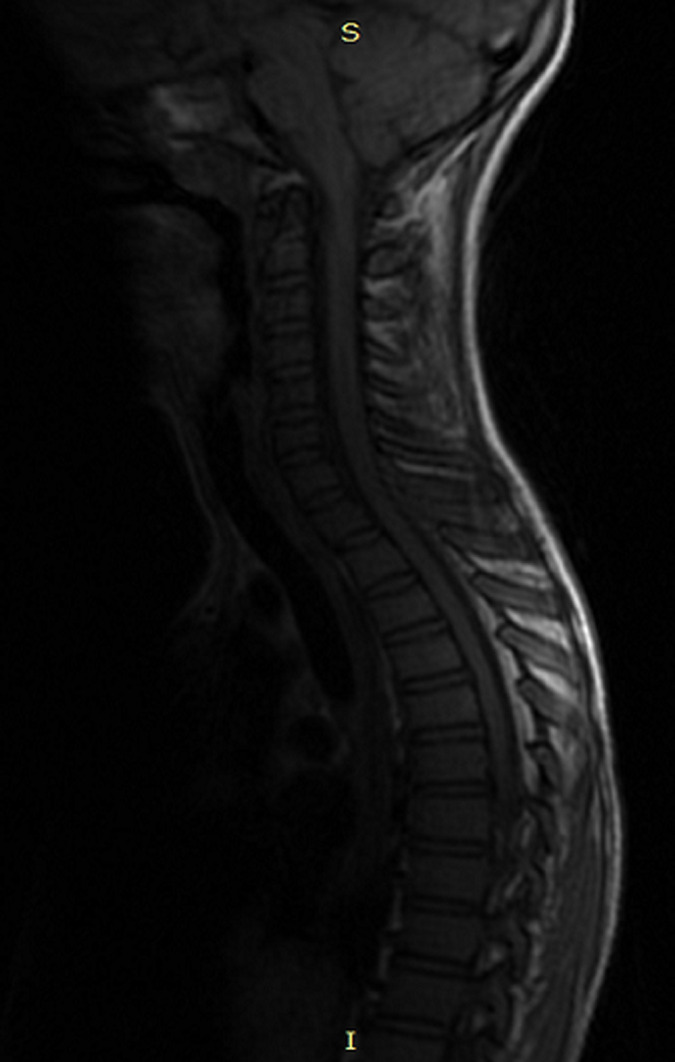
hypo signal of the inter-spinous space C7-D1

**Figure 2:**
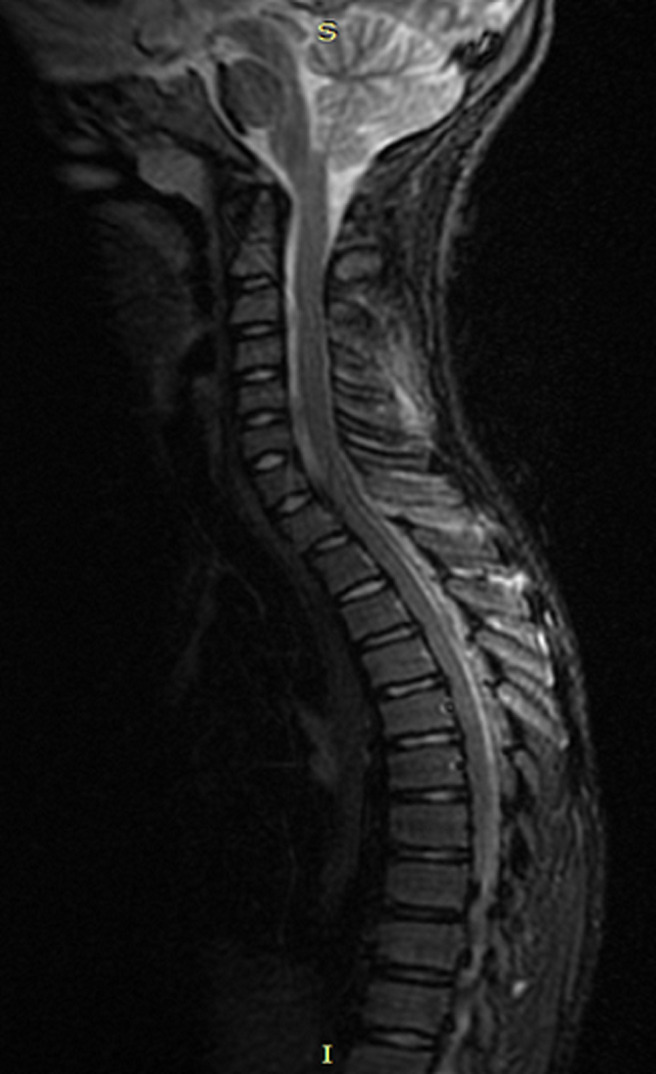
hyper signal T2 of the posterior arch of C7 and D1

**Figure 3:**
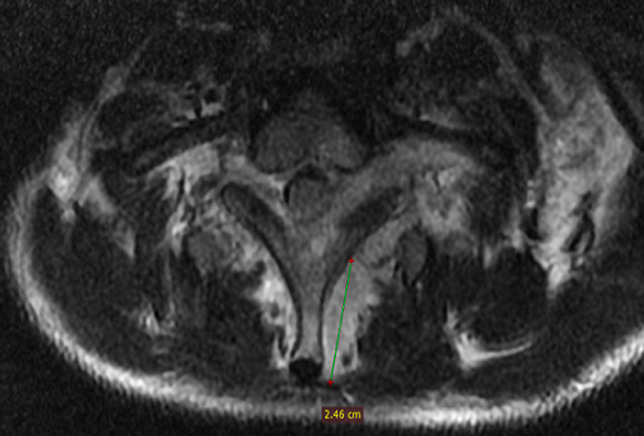
signal abnormality associated with posterior unilamellar periosteal reaction and linear hypo signal T1 of the cortical endo canal of the posterior arch of D1

A 12-year old child consults for febrile torticollis, without alteration of the general condition, and the evolution was for three weeks. The medical history of the patient does not find any pathological history, and he was well vaccinated. There was no tuberculosis notion of contagion. Physical exam found torticollis with mild pain in palpation in the lower cervical spine, while there were no swell and the temperature was 38~38.5°, we noted a biological inflammatory syndrome (WB: 16,000 mg/L; SR: 70 mg/L; CRP: 200 mg/L). The tuberculin skin test was positive, and search of tuberculosis (TB) in sputum and urine was negative. X-ray of the cervical spine was normal. More investigation with magnetic resonance imaging (MRI) was done and showed: signal abnormality of the cancellous bone of the posterior arch of C7 and D1, extended to the back of the vertebral body such as hyposignal in T1 (Figure 1), a discreet hyper signal in T2 (Figure 2), taking contrast after gadolinium injection. This signal abnormality is associated with posterior unilamellar periosteal reaction of the side of the left vertebral lamina and linear hypo signal T1 of the cortical endo canal of the posterior arch of D1 and C7 with the presence of some small erosion (Figure 3). The diagnosis was in favor of a specific infection, a radiological biopsy was not able for a technical reason, so we decided to do a surgical biopsy. In per operative, we found a collection in the interspinous space. The histological exam confirmed tuberculosis disease. The patient had tuberculosis chemotherapy for 12 months and immobilization with minerva. At the follow up of 3 years, we note a full motion of the cervical spine without any pain or any sign of recurrence.

## Discussion

Spinal tuberculosis is the most common localization of extrapulmonary tuberculosis. Its frequency is estimated to 2% of all localization and 50% of osteoarticular tuberculosis [1,2]. It mainly affects the thoracolumbar spine; the involvement of the lower cervical spine is estimated between 3% and 5% [1,3,4]. Posterior arch involvement is by contiguity in 90%, isolated involvement is rarer estimated to 1 to 3% of spinal tuberculosis [2-4]. Physical examination is poor and mainly consists of neck pain, signs of tuberculosis impregnation are not constant, and in late evolution neurological compression may be found [3-5]. The tuberculin skin test is contributive in non vaccinated patient. A biological inflammatory syndrome is frequent with high C-reactive protein (CRP). Radiological investigations are primordial, X-ray can demonstrate osteolytic lesion in the posterior arch, but these lesions can be difficult to demonstrate, in early manifestation and due to the inter-position of the anatomic element. More investigation helps to diagnose, computed tomography (CT) scan represents a good alternative to study the posterior arch and show the osteolytic lesion. MRI allows a multiplanar analysis of lesions, a study of the spinal cord, shows lesions detected at an early stage of granuloma without lysis, it also analyses the soft tissue [2,3,6,7]. Histological confirmation of the diagnosis of tuberculosis is mandatory, biopsy with CT scan tends to be the reference procedure. But, if it may not be possible, the surgical approach should be performed. Medical treatment should be initiated early and duration is 9-12 months [8,9]. Our patient had a surgical biopsy, which confirmed the diagnosis. She had the TB chemotherapy for the appropriate period, with orthopaedic immobilisation. In the final follow up the clinical and radiological investigations have not shown any sign of recurrence.

## Conclusion

The cervical spine is a rare localization of tuberculosis. The apophysis localization is more uncommon, the clinical presentation is poor especially in children. Neck pain or torticollis with fever must be investigated. MRI is the Gold standard for the study of the spine. The histological examination is mandatory for the diagnosis. The management bases on tuberculosis chemotherapy and immobilization to prevent instability.
